# Dental Practitioners’ Knowledge and Attitudes Toward the Etiology, Diagnosis, and Treatment of Peri-Implantitis

**DOI:** 10.3390/dj12120387

**Published:** 2024-11-27

**Authors:** Osama Zakaria, Afsheen Tabassum, Dina Attia, Turki Alshehri, Danya A. Alanazi, Jana Alshehri, Sami Alshehri, Aditi Chopra, Marwa Madi

**Affiliations:** 1 Department of Biomedical Dental Sciences, College of Dentistry, Imam Abdulrahman Bin Faisal University, P.O. Box 1982, Dammam 31441, Saudi Arabia; oazakaria@iau.edu.sa (O.Z.); smalshehri@iau.edu.sa (S.A.); 2 Department of Preventive Dental Sciences, College of Dentistry, Imam Abdulrahman Bin Faisal University, P.O. Box 1982, Dammam 31441, Saudi Arabia; atabassum@iau.edu.sa; 3 Department of Pediatric Dentistry and Dental Public Health, Faculty of Dentistry, Alexandria University, Alexandria 21527, Egypt; dina.ali@alexu.edu.eg; 4 Department of Substitutive Dental Sciences, College of Dentistry, Imam Abdulrahman Bin Faisal University, P.O. Box 1982, Dammam 31441, Saudi Arabia; taalshehri@iau.edu.sa; 5 College of Dentistry, Imam Abdulrahman Bin Faisal University, P.O. Box 1982, Dammam 31441, Saudi Arabia; 2210000386@iau.edu.sa (D.A.A.); 2210000199@iau.edu.sa (J.A.); 6 Department of Periodontology, Manipal College of Dental Sciences, Manipal, Manipal Academy of Higher Education, Manipal, Karnataka, India 576104, India; aditi.chopra@manipal.edu

**Keywords:** peri-implantitis, dental implant, education, knowledge, awareness, etiology, diagnosis, treatment

## Abstract

**Objectives:** The objective of this study was to assess the level of knowledge and attitude about the etiology, diagnosis, and management of peri-implantitis among dental practitioners. **Methods:** An online cross-sectional study on 303 dentists in Saudi Arabia was conducted. A closed-ended survey consisting of 28 questions was designed. Three sections were created, i.e., (1) participants’ characteristics; (2) knowledge of peri-implantitis etiology, risk factors, diagnosis, and complications; and (3) the use of antibiotics to manage peri-implantitis and methods used for diagnosis and treatment. The data were analyzed using mean and percentages. **Results:** A total of 303 dentists participated in this study with a 78.8% response rate, comprising 80% general dentists and 20% specialists. The majority of dentists demonstrated knowledge about peri-implantitis, with 85.5% identifying bacterial plaque as an etiologic factor and 83.8% recognizing it as an inflammatory reaction. Regarding risk factors, 88.1% identified smoking and 86.1% recognized periodontitis as contributors to peri-implant disease development. In terms of clinical decision making, 63% of participants considered implant mobility as a definitive indication for implant removal, and more than half reported using crater-like bone defects surrounding implants as a diagnostic criterion for peri-implantitis. For antimicrobial therapy, amoxicillin alone (30%) was the most commonly prescribed antibiotic, followed by the combination of amoxicillin with metronidazole (29%). Despite these treatment approaches, it is important to note that currently there are no established, predictable protocols for treating any phase of peri-implant disease. **Conclusion:** While participating dentists demonstrated a strong understanding of peri-implantitis etiology and risk factors, particularly regarding bacterial plaque and smoking as major contributors, there was considerable variation in antibiotic selection and usage patterns. These findings suggest the need for standardized guidelines and further research to establish evidence-based protocols for managing peri-implant diseases.

## 1. Introduction

Dental implants are considered as the optimal alternative option to replace missing teeth [1]. Biological complications affecting osseointegrated implants are a major concern in contemporary dentistry. These complications primarily involve inflammatory conditions triggered by bacterial insults [1,2,3]. There are two distinct clinical types: peri-implant mucositis and peri-implantitis. Both conditions feature inflammatory lesions; however, only peri-implantitis is characterized by the loss of supporting bone [4]. Healthy peri-implant tissue often exhibits no signs of inflammation symptoms (redness, swelling, or profuse bleeding on probing) and an absence of further bone loss after initial healing or an increase in probing depth [5]. However, the clinical diagnosis of peri-implantitis is made by assessing the presence of bone loss after initial healing; presence of peri-implant inflammation as measured by redness, swelling, or profuse bleeding on probing [6]; and greater probing depth in comparison to the probing depth following the placement of the prosthetic reconstruction. A radiographic bone level of ≥3 mm combined with BOP and PD of ≥6 mm is suggestive of peri-implantitis in the absence of prior radiographs [5].

The definition and diagnosis of peri-implant diseases have evolved over time, with varying criteria proposed in the literature. According to the consensus report of Workgroup 4 of the 2017 World Workshop, peri-implantitis diagnosis without baseline information requires a probing depth ≥6 mm, presence of bleeding and/or suppuration on gentle probing, and bone level ≥3 mm apical to the most coronal portion of the implant or at the rough–smooth interface in tissue-level implants [7]. However, alternative case definitions have been proposed, with some authors suggesting different thresholds such as progressive marginal bone loss ≥2 mm combined with probing depth ≥5 mm and bleeding/suppuration on probing [8]. More recent approaches have advocated for the implementation of staging and grading systems that consider risk factors and progression rates [9], while others have highlighted the variations in diagnostic thresholds for probing depth and bone loss measurements [5,10,11,12]. This diversity in case definition reflects the evolving nature of our understanding of peri-implant diseases and emphasizes the need for standardized diagnostic criteria. Since implant loss could occur from untreated peri-implantitis, it is thought to be the most difficult biological consequence [1,5,13].

While peri-implant mucositis may be present before the development of peri-implantitis [14], the presence of mucositis does not necessarily lead to progression to peri-implantitis [7]. These conditions, though related, represent distinct pathological entities with their own specific characteristics and risk factors [5]. The understanding of peri-implant diseases presents significant challenges in contemporary implant dentistry. The exact pathogenesis remains poorly understood [5], and while various factors have been associated with these conditions, definitive causative relationships have yet to be established [7]. This uncertainty extends to treatment approaches, where current interventions show unpredictable and often poor long-term outcomes [15].

However, for peri-implant diseases to be potentially treated, dentists should have adequate knowledge and training regarding the various diagnostic methods and treatment approaches [16]. Previous studies [16,17] have determined the knowledge and awareness regarding peri-implant disease among dentists and found that dentists appear to have a sufficient level of awareness and knowledge regarding implant procedures and their associated complications [17]. Younger practitioners were found to have more up-to-date information and understanding regarding implant therapy than older practitioners [17]. Therefore, it necessary to provide regular training to all dentists who place dental implants on a regular basis with basic education on periodontal and peri-implant disease [18,19].

While the most effective peri-implantitis treatment remains unidentified [20], systemic antibiotics as adjuncts to mechanical debridement and/or surgical procedures have shown some promise in treating peri-implantitis [21,22]. However, significant variation exists in antibiotic prescribing practices among dental practitioners, with no clear consensus on protocols [23], and prescribing patterns vary widely across different countries and clinical settings [24]. Despite being commonly recommended in treatment protocols, similar to periodontitis treatment approaches [25], the use of systemic antibiotics in peri-implantitis treatment remains controversial due to limited scientific evidence [21] and increasing global concerns about antibiotic resistance, leading some researchers to conclude that their use cannot be justified as a part of standard treatment protocols [26]. This knowledge gap directly impacts patient care and needs to be assessed.

The understanding of peri-implant diseases presents significant challenges in contemporary implant dentistry. The exact pathogenesis remains poorly understood [5], and while various factors have been associated with these conditions, definitive causative relationships are yet to be established [7].

There are limited number of studies addressing the knowledge and treatment of peri-implant disease in Saudi Arabia, with existing research primarily focused on the central region [27]. Moreover, it is crucial to assess the level of understanding among dentists in private practice, not just those working in academic settings.

Therefore, the aim of the present study was to assess the level of knowledge, attitudes, and practice of general and specialist dentists in Saudi Arabia regarding the causes, symptoms, and treatment of peri-implantitis.

## 2. Materials and Methods

### 2.1. Study Design

This cross-sectional study was carried out in the Eastern province of Saudi Arabia using Google Forms (Google, Inc., Mountain View, CA, USA), a web-based survey instrument. The current study was approved by the institutional review board (IRB) at Imam Abdulrahman bin Faisal University, Saudi Arabia, Dammam (IRB-2022-02-353). Self-administered validated questionnaire from the previous study [19] was distributed through social media channels (Twitter, WhatsApp, and Instagram) during the period from September 2023 to December 2023.

A total of 380 interns, residents, and dentists were invited to participate in this study. All participants provided written informed consent for the use of their data. Furthermore, the study ensured the confidentiality of the participants’ information by maintaining their anonymity. To avoid numerous entries from the same participant, the response set was set to only one response. All incomplete surveys were not included in the statistical analysis.

### 2.2. Sample Size Determination

The sample size was estimated using a 95% confidence level (α = 0.05), a 5% confidence interval, and statistical power of 0.80, which determined that 385 participants were required. A convenience sampling method was adopted, and the questionnaire was distributed via registered student emails and WhatsApp.

Study instruments and variables:

A previously validated questionnaire served as the basis for the creation of a 28-item closed-ended survey. Three sub-divisions were formed in the questionnaire: (1) knowledge about the etiology, risk factors, diagnosis, and complications of peri-implantitis; (2) attitudes toward diagnosis and treatment methods; and (3) usage of antibiotics in managing peri-implantitis. These factors comprise the first three characteristics of the survey participants (gender, whether they assist in implant surgery, and attend educational training about dental implants and peri-implantitis). To verify the validity and simplicity of the questionnaire, a draft was sent to eight dental implantology experts at the College of Dentistry, IAU. These professionals provided feedback on the questionnaire’s content domains, and the final analysis did not include their responses. Subsequently, a web-based pilot study was conducted on 20 participants to evaluate the readability, simplicity, and average time required to complete the task.

The data were analyzed using the Statistical Package for Social Sciences (SPSS) version 22 (IBM Corp., Armonk, NY, USA). Correct answers in each section were defined, and descriptive statistics (counts and percentages) were calculated.

## 3. Results

A total of 303 out of 385 participants participated in this study, resulting in a response rate of 78.8%. Over half the dentists were males (52.8%), the majority had a bachelor’s degree (81.2%) as their highest degree attained, and 111 dentists (36.6%) worked at dental college as general dentists. A total of 198 participants (65.3%) did not perform or assist in implant surgery on a regular basis, whereas more than half of the dentists (56.4%) received some kind of formal education about implant therapy (58.1%) (Table 1).

**Figure 1:**
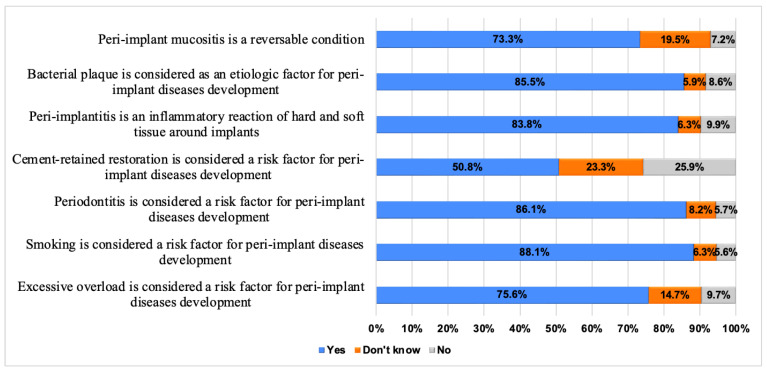
Knowledge of graduated dentists about peri-implantitis etiology and risk factors.

Knowledge of graduated dentists about peri-implantitis etiology and risk factors is shown in Figure 1. The majority of dentists (85.5%) were knowledgeable about bacterial plaque being an etiological factor for peri-implant diseases, and that peri-implantitis is an inflammatory reaction (83.8%). In regard to the risk factors, the majority of the participants (88.1%) identified smoking as a risk factor, followed by periodontitis (86.1%), and then excessive implant overloading (75.6%).

Table 2 shows the knowledge of the participants about diagnosing peri-implantitis. Regarding the signs and symptoms that help dentists to diagnose peri-implantitis, bleeding/suppuration upon gentle probing with a blunt instrument was reported by 54.1%. Most of the participants (73.9%) reported that they always used peri-apical radiograph after implant placement, and a slightly higher percentage (75.9%) reported performing the radiograph immediately after the implant placement (always). In addition, more than half the participants (59.7%) reported that they always rely on vertical bone loss with a saucer-shaped defect as a radiographic representation of peri-implantitis.

**Figure 2:**
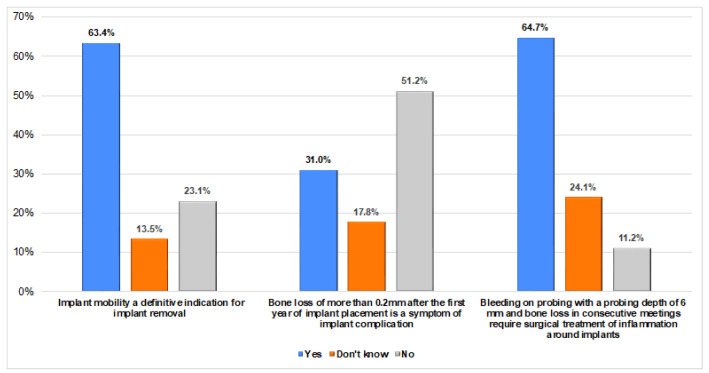
Knowledge of graduated dentists about peri-implantitis manifestations.

Regarding the knowledge about peri-implantitis manifestations, 63.4% considered implant mobility as a definitive indicator for implant removal, and 64.7% considered bleeding on probing with a probing depth of 6 mm and bone loss around implant require surgical treatment (Figure 2).

The knowledge about peri-implantitis treatment instruments and methods is shown in Table 3. Overall, 46.9% of the participants reported always using plastic curettes and 76.2% reported always using oral hygiene instructions as the method for the treatment of peri-implantitis.

**Figure 3:**
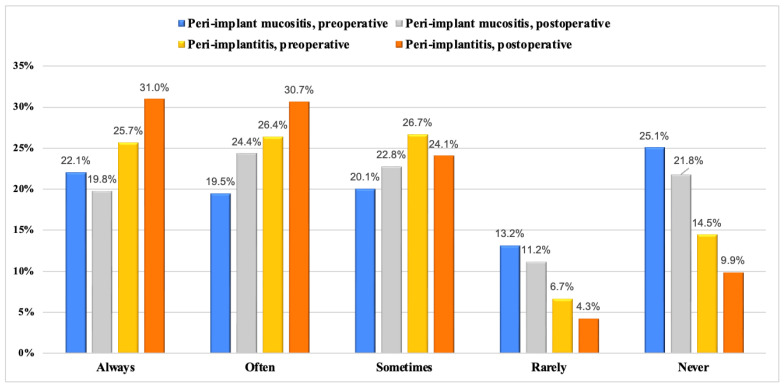
Frequency of peri- and post-operative use of systemic antibiotics for treatment of peri-implant mucositis and peri-implantitis among graduated dentists.

**Figure 4:**
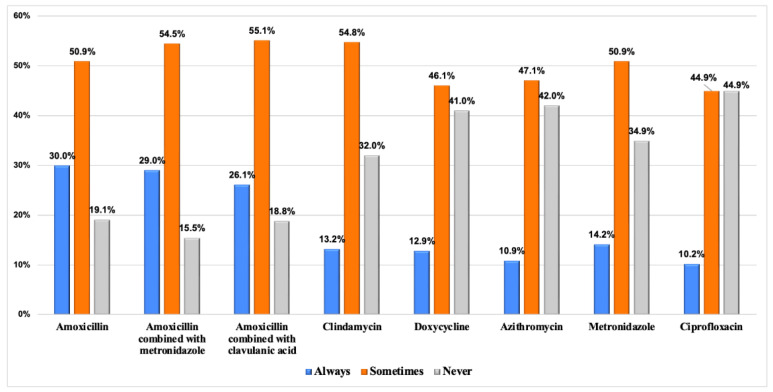
Frequency of the antibiotic regimen used by graduated dentists for the management of peri-implant diseases.

The use of systemic antibiotics for treating peri-implant diseases is shown in Figure 3 and Figure 4. Figure 3 provides insights into the utilization of antibiotics in the context of peri-implant mucositis and peri-implantitis, peri-operative (at the time of treatment) and post-operative (after treatment) categorized by the frequency of usage. Peri-operative antibiotic utilization for peri-implantitis shows rates from 25.7% (Always) to 14.5% (Never), while post-operative rates range from 31.0% (Always) to 9.9% (Never). Among the antibiotics studied, amoxicillin, amoxicillin with metronidazole, and amoxicillin with clavulanic acid were used with varying frequencies, ranging from 26.1% to 30.0% (Always), 50.9% to 54.5% (Sometimes), and 15.5% to 19.1% (Never).

## 4. Discussion

Given the pivotal role of implant treatment in dentistry, the escalating challenge of peri-implantitis is a great concern for dentists worldwide. Estimating the prevalence of peri-implantitis poses difficulties due to probable regional disparities, diverse case definitions of peri-implantitis, and variations in follow-up time points [28]. A systematic review and meta-analysis found that the prevalence of peri-implantitis at the implant level was 9.25%, while at the subject-level, the prevalence was 19.83%. Furthermore, the peri-implant mucositis prevalence at the implant level was calculated to be 29.48%, with a subject-based prevalence of 46.83% [28]. Another cross-sectional study conducted over 657 implants reported that, at the patient level, peri-implant mucositis and peri-implantitis were found to be present in 66.5% and 15% cases, while at the implant level, the prevalence was 62.6% and 7.5% [29]. A study conducted in Riyadh, Saudi Arabia, revealed that peri-implant mucositis prevalence both at the implant and subject levels (51% and 43%) was significantly higher than peri-implantitis (22% and 27%) in the Saudi population [30]. These findings highlight the importance of understanding the etiology and making an accurate diagnosis and treatment plan of peri-implantitis amongst dental practitioners in Saudi Arabia.

The high level of understanding among dentists (85.5%) about microbial plaque as an etiologic factor in peri-implant infections suggests that the role of plaque in such diseases is well established [31,32,33] and disseminated in dental education and the current literature [5,34]. Our results are in accordance with Tripathi et al. [17] who reported that most dentists (80.3% with masters and 81.6% who attended implantology courses) recognized bacterial plaque as the major etiological factor of peri-implant disease. Likewise, in the current study, the recognition by 83.8% of dentists that the inflammatory nature of the peri-implant diseases indicates a broad consensus on the pathophysiology of peri-implantitis [14].

Smoking was recognized by 88.1% of dentists as a risk factor for peri-implant infection that exhibits a strong awareness of the systemic factor’s influence on oral health, particularly peri-implant disease development. In addition, a history of periodontitis was recognized by 86.1% of participants as a risk factor. These findings are consistent with a study of Australian periodontists, which identified bacterial plaque, smoking, and past periodontitis as the critical elements contributing to peri-implantitis [35]. Furthermore, surveys of American and European periodontists have shown that both groups consider a history of periodontitis as well as smoking as the most significant risk factors [36]. These findings are aligned with many previous studies [5,37,38,39].

Many participants (75.6%) recognized excessive overloading as a risk factor for peri-implant infections. In a systematic review, Bertolini et al. investigated the association between traumatic/excessive occlusal forces and bone loss around implants. The authors found that, based on four animal studies, lower levels of occlusal loading were not linked to bone loss around implants [40]. In contrast to our results, Tripathi et al. reported that only 38.5% of the participants identified adverse loading as a potential risk factor for peri-implantitis [17]. Current evidence does not support occlusal overload as an etiologic factor for peri-implant diseases [41]. While biomechanical factors play a role in implant success, well-designed clinical studies have failed to demonstrate a direct causal relationship between occlusal overload and peri-implantitis [42,43]. The primary etiology remains bacterial in nature, with various other risk factors such as smoking and history of periodontitis playing more significant roles in disease development [5].

However, nearly half of the survey respondents (50.8%) were unaware of cement-retained restorations as potential contributors to peri-implant disease. Despite meticulous clinical management, cementation of these restorations carries the chance of retaining extra cement in the peri-implant area. This excess cement can serve as a hub for oral microorganism colonization, leading to the formation of biofilms that may result in peri-mucositis or peri-implantitis [44,45]. This knowledge gap suggests a need for further education regarding the impact of prosthetic choices and their management for preventing peri-implant diseases. The impact of prosthetic design on peri-implant health has gained increasing attention in the recent literature. Particularly, the restoration emergence profile has been identified as a significant factor influencing marginal bone stability. Studies have demonstrated that restoration emergence angle shows a positive correlation with marginal bone loss [46,47] with implant restorations having emergence angles ≥30° and showing significantly greater marginal bone loss [48,49]. Additionally, prosthetic factors such as excess cement retention, inadequate emergence profiles, and over contoured restorations may compromise access for proper oral hygiene and create environments conducive to plaque accumulation [50]. The design of the prosthetic component can also affect the distribution of mechanical stress to the surrounding bone, with excessive off-axis loading potentially contributing to bone remodeling [51]. Furthermore, screw-retained restorations have shown advantages over cement-retained restorations in terms of retrievability and management of biological complications, suggesting their potential benefit in preventing and managing peri-implant diseases [52].

The dentists reported in the present study that they utilize specific signs and symptoms for establishing the diagnosis of peri-implantitis, including bleeding on probing (BoP; 54.1%), crater-like bone defects (54.1%), and periodontal probe advancement of 5 mm or more into the sulcus (50.2%). Additionally, the majority of the participants indicated that they always relied on peri-apical radiographs post-implant placement, particularly immediately after placement and at prosthesis delivery. Furthermore, the saucer-shaped vertical bone defect was commonly cited as a radiographic indicator of peri-implantitis in the diagnostic process (59.7%). These findings align with the existing literature on peri-implantitis diagnosis, which emphasizes the importance of clinical examination (such as BoP, suppuration, and periodontal probing depth) and radiographic assessments for identifying peri-implant bone loss around implants [7,10]. The diagnostic criteria for peri-implantitis is specified as a pocket depth of ≥6 mm, BOP or suppuration, and a bone loss of ≥3 mm, if there are no previous records of the patient present [15]. Additionally, our results are in agreement with a study conducted on New Zealand specialists that investigated the understanding, diagnosis, and management of peri-implantitis and found that the criteria used by the participants to diagnose peri-implantitis was increased probing depths and radiographic evidence of bone loss [53].

The knowledge of graduated dentists about using titanium and plastic curettes, stainless steel instrument, and laser as well as the treatment options for peri-implantitis exhibited that almost half the participants (46.9%) reported always using plastic curettes and 34.3% preferred titanium curettes. Our findings closely align with previous findings in which 48.1% of the participants preferred using plastic curettes and only 17.6% favored titanium curettes [17]. However, Alqahtani et al. [27] reported a smaller proportion of dentists, andonly 37.2%, considered plastic curettes to be the optimal instrument for implant surface scaling. These results underscore the ongoing preference of dentists for less abrasive debridement tools around implants.

In the present study, most of the dentists reported oral hygiene education (76.2%), non-surgical debridement (54.1%), and antimicrobial mouthwash or gel (51.7%) and chlorohexidine (35.3%) as a treatment strategy for peri-implant infections. However, in the literature, no consensus could be observed on the most efficient treatment method [54]. The management of peri-implant diseases remains a significant clinical challenge, with no universally accepted or predictable treatment protocols established to date [5]. While various therapeutic approaches have been proposed, including mechanical debridement, surgical interventions, and adjunctive treatments, their long-term efficacy remains questionable [15]. Non-surgical approaches are considered primarily as they can potentially decrease pocket depth by approximately 1 mm and result in a reduction in BoP [55]. However, in advanced cases, these non-surgical treatments cannot prevent the progression of the disease and thorough elimination of the bacteria, particularly around the rough surfaces of implants [55,56]. To augment mechanical debridement, adjunctive therapies like local antiseptics [57], probiotics [58], antibiotics [59], and lasers [60] have been recommended. Nonetheless, the current research shows that these additional interventions offer limited extra benefits in improving clinical outcomes. In contrast, surgical interventions have proved to be more efficient, resulting in reduced BoP, shallower probing depths, and gains in radiographic bone levels [61]. Systematic reviews have shown that most current treatments, at best, may only slow disease progression rather than achieve complete resolution [8]. Notably, controlled studies have demonstrated that systemic antibiotic therapy, despite its widespread use, lacks strong evidence supporting its effectiveness in treating peri-implant diseases [62]. This therapeutic uncertainty is further complicated by the complex nature of peri-implant disease progression and the variety of implant surfaces and configurations available [7].

In cases of peri-implant mucositis, 22% of participants opted for pre-operative antibiotics and 19.8% prescribed them post-operatively. In contrast, for peri-implantitis, preoperative antibiotic use was reported at 25.7%, with post-operative usage at 31.0% among participants. Amoxicillin was the most prescribed (30.0%), followed by amoxicillin in combination with metronidazole (29.0%). Alqahtani et al. [27] revealed that 76% of dentists viewed amoxicillin–metronidazole as the preferred antibiotic regimen, with only 5% selecting ciprofloxacin 36. These results also align with the observations of Polymeri et al. [36] who noted less frequent use of antiseptics and antibiotics among dentists in Europe and the United States compared to other treatment modalities. No clear consensus on protocols and prescribing patterns of antibiotics for peri-implantitis treatment exist due to limited scientific evidence [21,23].

The findings of our study should be interpreted within the context of current limitations in peri-implant disease management. While various treatment approaches are being utilized in clinical practice, the literature demonstrates consistently unpredictable and often poor long-term outcomes [15]. Furthermore, intervention studies frequently show limited success in achieving complete disease resolution [14], and the lack of standardized, evidence-based protocols [8] makes it difficult to compare treatment outcomes across studies. Therefore, while our findings provide insights into current clinical practices, they also emphasize the pressing need for high-quality research to develop more predictable therapeutic approaches and establish evidence-based treatment guidelines.

The main limitation of the present study is that the data were collected only from the Eastern province of Saudi Arabia and findings cannot be generalized for all dentists across the country. The sample size used in the study was not large enough to provide a comprehensive understanding of dentists’ knowledge and awareness of peri-implant infections. In addition, cross-sectional surveys rely on self-reported data from participants, which may introduce response bias or social desirability bias [63]. Also, there is a lack of inferential statistical analyses to examine potential associations and differences between demographic variables and clinical practices. Patterns in gender-based differences in management approaches, associations between years of experience and treatment preferences, and variations between general practitioners and specialists would have provided deeper insights into factors influencing clinical decision making and knowledge levels among dental practitioners. Future research should incorporate comprehensive statistical analyses to investigate these potential associations and their implications for dental education and clinical practice guidelines.

## 5. Conclusions

The study revealed a high level of knowledge and awareness among dental practitioners in Saudi Arabia regarding the etiology, risk factors and diagnosis of peri-implantitis. The majority recognized bacterial plaque as a key etiologic factor and smoking as a significant risk factor. While most participants accurately identified implant mobility as a sign for implant removal, there were variations in diagnostic approaches, with a preference for using crater-like bone defects as an indicator of peri-implantitis. Regarding treatment approaches, although there is currently no consensus on antibiotic protocols and prescribing patterns for peri-implantitis management, this study found amoxicillin to be the most frequently prescribed antibiotic among practitioners. Future research is warranted to explore various therapeutic approaches and develop evidence-based treatment guidelines for peri-implantitis management.

## Figures and Tables

**Table 1:** Sample characteristics (*n* = 303).

Variable	Frequency *n* (%)	
Gender	Males	160 (52.8)
	Females	143 (47.2)
Highest degree attained	Bachelor’s degree	246 (81.2)
	Master’s degree	26 (8.6)
	Fellowship	14 (4.6)
	Ph.D.	17 (5.6)
Work sector	Dental college	111 (36.6)
	Private sector	105 (34.7)
	Governmental sector	87 (28.7)
Perform or assist in implant surgery on a regular basis	Yes	105 (34.7)
	No	198 (65.3)
Received formal education/training on implant therapy	Yes	171 (56.4)
	No	132 (43.6)
Attended lecture on diagnosis/treatment of peri-implantitis	Yes	127 (41.9)
	No	176 (58.1)

**Table 2:** Knowledge of graduated dentists about diagnosing peri-implantitis.

		Relative Use *n* (%)				
		Always	Often	Sometimes	Rarely	Never
What signs and symptoms help diagnose peri-implantitis?	Bleeding/suppuration upon gentle probing with a blunt instrument	164 (54.1)	85 (28.1)	37 (12.2)	11 (3.6)	6 (2.0)
	Marginal tissue swollen and red	135 (45.6)	92 (30.4)	58 (18.1)	7 (2.3)	11 (3.6)
	Periodontal probe can be advanced ≥5 mm into the sulcus	152 (50.2)	97 (32.0)	37 (12.2)	9 (3.0)	8 (2.6)
	Pain	118 (38.9)	98 (32.3)	64 (21.2)	13 (4.3)	10 (3.3)
	Crater-like bone defect around implant	164 (54.1)	94 (31.0)	33 (10.9)	3 (1.0)	9 (3.0)
Radiographs you perform after implant placement	Panoramic radiograph	118 (38.9)	51 (16.8)	70 (23.2)	34 (11.2)	30 (9.9)
	Peri-apical X-ray	224 (73.9)	26 (8.6)	38 (12.5)	9 (3.0)	6 (2.0)
	CBCT	88 (29.0)	48 (15.8)	99 (32.7)	42 (13.9)	26 (8.6)
When do you perform the baseline X-ray after implant placement?	Immediately after surgery	230 (75.9)	28 (9.2)	29 (9.6)	3 (1.0)	13 (4.3)
	After 1–3 weeks	81 (26.7)	73 (24.1)	73 (24.1)	34 (11.2)	42 (13.9)
	At implant uncovering	109 (36.0)	74 (24.2)	62 (20.5)	32 (10.6)	26 (8.7)
	At prostheses delivery	182 (60.1)	46 (16.2)	37 (12.2)	24 (7.9)	14 (3.6)
Radiographic representation of peri-implantitis you rely on in your diagnosis	A radio-opaque area at the apical aspect of the implant	42 (13.9)	51 (16.8)	52 (17.2)	42 (13.8)	116 (38.3)
	A radiolucent area at the apical aspect of the implant	113 (37.3)	76 (25.1)	66 (21.8)	18 (5.9)	30 (9.9)
	Vertical bone loss with saucer-shaped defect	181 (59.7)	65 (21.5)	44 (14.5)	3 (1.0)	10 (3.3)

**Table 3:** Knowledge of graduated dentists about treatment instruments and methods for peri-implantitis.

Treatment		Relative Use *n* (%)				
		Always	Often	Sometimes	Rarely	Never
What type of instruments/agents do you use for implant debridement?	Titanium curettes	104 (34.3)	47 (15.5)	54 (17.8)	22 (7.3)	76 (25.1)
	Plastic curettes	142 (46.9)	71 (23.5)	51 (16.8)	8 (2.6)	31 (10.2)
	Stainless steel instrument	40 (13.2)	50 (16.5)	55 (18.1)	26 (8.6)	132 (43.6)
	Laser	47 (15.5)	62 (20.5)	76 (25.1)	27 (8.9)	91 (30.0)
	Hydrogen peroxide	47 (15.5)	72 (23.8)	61 (20.1)	34 (11.2)	89 (29.4)
	Chlorhexidine	107 (35.3)	83 (27.4)	62 (20.5)	15 (4.9)	36 (11.9)
Which of the following treatment methods do you use for the treatment of peri-implantitis?	Oral hygiene instructions	231 (76.2)	34 (11.2)	25 (8.3)	1 (0.3)	12 (4.0)
	Antimicrobial gel OR mouthrinse	157 (51.7)	75 (24.8)	53 (17.5)	2 (0.7)	16 (5.3)
	Non-surgical debridement	164 (54.1)	62 (20.5)	54 (17.8)	4 (1.3)	19 (6.3)
	Local antibiotics	85 (28.1)	73 (24.1)	75 (24.8)	32 (10.6)	32 (12.4)
	Surgical debridement	66 (21.8)	57 (18.8)	92 (30.4)	36 (11.9)	52 (17.1)
	Resective surgery	44 (14.5)	48 (15.8)	82 (27.1)	51 (16.8)	78 (25.8)
	Regenerative surgery	51 (16.8)	58 (19.1)	79 (26.1)	44 (14.5)	71 (23.5)
	Control of occlusion	97 (32.0)	66 (21.8)	81 (27.0)	25 (8.3)	34 (11.2)

## Data Availability

Data will be provided upon request from the corresponding author.

## References

[1] Lang N.P., Berglundh T. (2011). Working Group 4 of the Seventh European Workshop on Periodontology. Periimplant Diseases: Where Are We Now?–Consensus of the Seventh European Workshop on Periodontology. J. Clin. Periodontol..

[2] Sanz M., Chapple I.L. (2012). Working Group 4 of the VIII European Workshop on Periodontology. Clinical Research on Peri-implant Diseases: Consensus Report of Working Group 4. J. Clin. Periodontol..

[3] Jepsen S., Berglundh T., Genco R., Aass A.M., Demirel K., Derks J., Figuero E., Giovannoli J.L., Goldstein M., Lambert F. (2015). Primary Prevention of Peri-implantitis: Managing Peri-implant Mucositis. J. Clin. Periodontol..

[4] Lindhe J., Meyle J., Group D of the European Workshop on Periodontology (2008). Peri-implant Diseases: Consensus Report of the Sixth European Workshop on Periodontology. J. Clin. Periodontol..

[5] Schwarz F., Derks J., Monje A., Wang H. (2018). Peri-implantitis. J. Clin. Periodontol..

[6] Rokaya D., Srimaneepong V., Wisitrasameewon W., Humagain M., Thunyakitpisal P. (2020). Peri-Implantitis Update: Risk Indicators, Diagnosis, and Treatment. Eur. J. Dent..

[7] Berglundh T., Armitage G., Araujo M.G., Avila-Ortiz G., Blanco J., Camargo P.M., Chen S., Cochran D., Derks J., Figuero E. (2018). Peri-implant Diseases and Conditions: Consensus Report of Workgroup 4 of the 2017 World Workshop on the Classification of Periodontal and Peri-Implant Diseases and Conditions. J. Periodontol..

[8] Ramanauskaite A., Becker K., Juodzbalys G., Schwarz F. (2018). Clinical Outcomes Following Surgical Treatment of Peri-Implantitis at Grafted and Non-Grafted Implant Sites: A Retrospective Analysis. Int. J. Implant Dent..

[9] Romandini M., Berglundh J., Derks J., Sanz M., Berglundh T. (2021). Diagnosis of Peri-implantitis in the Absence of Baseline Data: A Diagnostic Accuracy Study. Clin. Oral Implant. Res..

[10] Monje A., Caballé-Serrano J., Nart J., Peñarrocha D., Wang H., Rakic M. (2018). Diagnostic Accuracy of Clinical Parameters to Monitor Peri-implant Conditions: A Matched Case-control Study. J. Periodontol..

[11] Lombardo G., Signoriello A., Marincola M., Bonfante E.A., Díaz-Caballero A., Tomizioli N., Pardo A., Zangani A. (2023). Five-Year Follow-Up of 8 and 6 Mm Locking-Taper Implants Treated with a Reconstructive Surgical Protocol for Peri-Implantitis: A Retrospective Evaluation. Prosthesis.

[12] Coli P., Jemt T. (2021). Are Marginal Bone Level Changes around Dental Implants Due to Infection?. Clin. Implant Dent. Relat. Res..

[13] Berglundh T., Jepsen S., Stadlinger B., Terheyden H. (2019). Peri-implantitis and Its Prevention. Clin. Oral Implant. Res..

[14] Salvi G.E., Stähli A., Imber J., Sculean A., Roccuzzo A. (2023). Physiopathology of Peri-implant Diseases. Clin. Implant Dent. Relat. Res..

[15] Renvert S., Persson G.R., Pirih F.Q., Camargo P.M. (2018). Peri-implant Health, Peri-implant Mucositis, and Peri-implantitis: Case Definitions and Diagnostic Considerations. J. Clin. Periodontol..

[16] Kadkhodazadeh M., Hosseinpour S., Kermani M.E., Amid R. (2018). Knowledge and Attitude of Iranian Dentists towards Peri-Implant Diseases. J. Adv. Periodontol. Implant Dent..

[17] Tripathi R., Vasudevan S., Palle A.R., Gedela R.K., Punj A., Vaishnavi V. (2020). Awareness and Management of Peri-Implantitis and Peri-Mucositis among Private Dental Practitioners in Hyderabad-A Cross-Sectional Study. J. Indian Soc. Periodontol..

[18] Cheng C.-D., Tsai Y.-W.C., Cheng W.-C., Lin F.-G., Weng P.-W., Chen Y.-W., Huang R.-Y., Chen W.-L., Shieh Y.-S., Sung C.-E. (2023). The Referral Pattern and Treatment Modality for Peri-Implant Disease between Periodontists and Non-Periodontist Dentists. BMC Oral Health.

[19] Madi M., Tabassum A., Attia D., Al Muhaish L., Al Mutiri H., Alshehri T., Zakaria O., Aljandan B. (2024). Knowledge and Attitude of Dental Students Regarding Etiology, Diagnosis, and Treatment of Peri-implantitis. J. Dent. Educ..

[20] Esposito M., Grusovin M.G., Worthington H.V. (2012). Treatment of Peri-Implantitis: What Interventions Are Effective? A Cochrane Systematic Review. Eur. J. Oral Implant..

[21] Jan van Winkelhoff A. (2012). Antibiotics in the Treatment of Peri-Implantitis. Eur. J. Oral Implant..

[22] Heitz-Mayfield L.J.A., Mombelli A. (2014). The Therapy of Peri-Implantitis: A Systematic Review. Int. J. Oral Maxillofac. Implant..

[23] Becker K., Gurzawska-Comis K., Klinge B., Lund B., Brunello G. (2024). Patterns of Antibiotic Prescription in Implant Dentistry and Antibiotic Resistance Awareness among European Dentists: A Questionnaire-based Study. Clin. Oral Implant. Res..

[24] Rams T.E., Slots J. (2023). Antimicrobial Chemotherapy for Recalcitrant Severe Human Periodontitis. Antibiotics.

[25] Slots J. (2004). Update on Human Cytomegalovirus in Destructive Periodontal Disease. Oral Microbiol. Immunol..

[26] McFall R. (2021). Antimicrobial Resistance: Where Are We Now?. Br. Stud. Dr. J..

[27] Alqahtani A.R., Gufran K., Alqahtani A.M., Alazemi F.N., Alzahrani K.M. (2021). Evaluating the Knowledge of General Dentist Towards the Management of Peri-Implant Diseases: A Multi-Center, Cross-Sectional Study. Open Dent. J..

[28] Lee C.-T., Huang Y.-W., Zhu L., Weltman R. (2017). Prevalences of Peri-Implantitis and Peri-Implant Mucositis: Systematic Review and Meta-Analysis. J. Dent..

[29] Obreja K., Ramanauskaite A., Begic A., Galarraga-Vinueza M.E., Parvini P., Sader R., Schwarz F. (2021). The Prevalence of Peri-implant Diseases around Subcrestally Placed Implants: A Cross-sectional Study. Clin. Oral Implant. Res..

[30] AlGhamdi J., Shafik S., Al-Mashat H. (2017). Prevalence of Peri-Implant Diseases among Patients Received Dental Implants at Riyadh City, KSA. IJAR.

[31] Ferreira S.D., Silva G.L.M., Cortelli J.R., Costa J.E., Costa F.O. (2006). Prevalence and Risk Variables for Peri-implant Disease in Brazilian Subjects. J. Clin. Periodontol..

[32] Roos-Jansåker A., Lindahl C., Renvert H., Renvert S. (2006). Nine-to Fourteen-year Follow-up of Implant Treatment. Part II: Presence of Peri-implant Lesions. J. Clin. Periodontol..

[33] Konstantinidis I.K., Kotsakis G.A., Gerdes S., Walter M.H. (2015). Cross-Sectional Study on the Prevalence and Risk Indicators of Peri-Implant Diseases. Eur. J. Oral Implant..

[34] Heitz-Mayfield L.J.A., Salvi G.E. (2018). Peri-implant Mucositis. J. Clin. Periodontol..

[35] Khan A., Sharma D. (2020). Management of Peri-Implant Diseases: A Survey of Australian Periodontists. Dent. J..

[36] Polymeri A., Loos B.G., Aronovich S., Steigmann L., Inglehart M.R. (2022). Risk Factors, Diagnosis, and Treatment of Peri-implantitis: A Cross-cultural Comparison of US and European Periodontists’ Considerations. J. Periodontol..

[37] Saaby M., Karring E., Schou S., Isidor F. (2016). Factors Influencing Severity of Peri-implantitis. Clin. Oral Implant. Res..

[38] Papathanasiou E., Finkelman M., Hanley J., Parashis A.O. (2016). Prevalence, Etiology and Treatment of Peri-implant Mucositis and Peri-implantitis: A Survey of Periodontists in the United States. J. Periodontol..

[39] Schmidlin P.R., Sahrmann P., Ramel C., Imfeld T., Müller J., Roos M., Jung R.E. (2012). Peri-Implantitis Prevalence and Treatment in Implant-Oriented Private Practices: A Cross-Sectional Postal and Internet Survey. Schweiz. Monatsschrift Für Zahnmed..

[40] Bertolini M.M., Del Bel Cury A.A., Pizzoloto L., Acapa I.R.H., Shibli J.A., Bordin D. (2019). Does Traumatic Occlusal Forces Lead to Peri-Implant Bone Loss? A Systematic Review. Braz. Oral Res..

[41] Heitz-Mayfield L.J., Schmid B., Weigel C., Gerber S., Bosshardt D.D., Jönsson J., Lang N.P., Jönsson J. (2004). Does Excessive Occlusal Load Affect Osseointegration? An Experimental Study in the Dog. Clin. Oral Implant. Res..

[42] Graves C.V., Harrel S.K., Rossmann J.A., Kerns D., Gonzalez J.A., Kontogiorgos E.D., Al-Hashimi I., Abraham C. (2016). The Role of Occlusion in the Dental Implant and Peri-Implant Condition: A Review. Open Dent. J..

[43] Morton D., Gallucci G., Lin W., Pjetursson B., Polido W., Roehling S., Sailer I., Aghaloo T., Albera H., Bohner L. (2018). Group 2 ITI Consensus Report: Prosthodontics and Implant Dentistry. Clin. Oral Implant. Res..

[44] Korsch M., Walther W., Marten S.-M., Obst U. (2014). Microbial Analysis of Biofilms on Cement Surfaces: An Investigation in Cement-Associated Peri-Implantitis. J. Appl. Biomater. Funct. Mater..

[45] Korsch M., Obst U., Walther W. (2014). Cement-associated Peri-implantitis: A Retrospective Clinical Observational Study of Fixed Implant-supported Restorations Using a Methacrylate Cement. Clin. Oral Implant. Res..

[46] Pascual R.L. (2022). Influence of Prosthetic and Surgical Parameters on Peri-Implant Marginal Bone Loss. Master’s Thesis.

[47] Kou Y., Li Q., Tang Z. (2023). Prosthetic Emergence Angle in Different Implant Sites and Their Correlation with Marginal Bone Loss: A Retrospective Study. J. Dent. Sci..

[48] Yi Y., Koo K., Schwarz F., Ben Amara H., Heo S. (2020). Association of Prosthetic Features and Peri-implantitis: A Cross-sectional Study. J. Clin. Periodontol..

[49] Wang L., Wang T., Lu Y., Fan Z. (2021). Comparing the Clinical Outcome of Peri-Implant Hard and Soft Tissue Treated with Immediate Individualized CAD/CAM Healing Abutments and Conventional Healing Abutments for Single-Tooth Implants in Esthetic Areas Over 12 Months: A Randomized Clinical Trial. Int. J. Oral Maxillofac. Implant..

[50] Wilson T.G. (2009). The Positive Relationship between Excess Cement and Peri-implant Disease: A Prospective Clinical Endoscopic Study. J. Periodontol..

[51] Katafuchi M., Weinstein B.F., Leroux B.G., Chen Y., Daubert D.M. (2018). Restoration Contour Is a Risk Indicator for Peri-implantitis: A Cross-sectional Radiographic Analysis. J. Clin. Periodontol..

[52] Wittneben J., Joda T., Weber H., Brägger U. (2017). Screw Retained vs. Cement Retained Implant-supported Fixed Dental Prosthesis. Periodontol. 2000.

[53] Russell A.A., Tawse-Smith A., Broadbent J.M., Leichter J.W. (2014). Peri-Implantitis Diagnosis and Treatment by New Zealand Periodontists and Oral Maxillofacial Surgeons. N. Z. Dent. J..

[54] Garaicoa-Pazmino C., Sinjab K., Wang H.-L. (2019). Current Protocols for the Treatment of Peri-Implantitis. Curr. Oral Health Rep..

[55] Renvert S., Hirooka H., Polyzois I., Kelekis-Cholakis A., Wang H.-L., Working Group 3 (2019). Diagnosis and Non-Surgical Treatment of Peri-Implant Diseases and Maintenance Care of Patients with Dental Implants—Consensus Report of Working Group 3. Int. Dent. J..

[56] Persson G.R., Samuelsson E., Lindahl C., Renvert S. (2010). Mechanical Non-Surgical Treatment of Peri-Implantitis: A Single-Blinded Randomized Longitudinal Clinical Study. II. Microbiological Results. J. Clin. Periodontol..

[57] Menezes K.M., Fernandes-Costa A.N., Silva-Neto R.D., Calderon P.S., Gurgel B.C.V. (2016). Efficacy of 0.12% Chlorhexidine Gluconate for Non-Surgical Treatment of Peri-Implant Mucositis. J. Periodontol..

[58] Amato M., Di Spirito F., D’Ambrosio F., Boccia G., Moccia G., De Caro F. (2022). Probiotics in Periodontal and Peri-Implant Health Management: Biofilm Control, Dysbiosis Reversal, and Host Modulation. Microorganisms.

[59] Shibli J.A., Ferrari D.S., Siroma R.S., de Figueiredo L.C., de Faveri M., Feres M. (2019). Microbiological and Clinical Effects of Adjunctive Systemic Metronidazole and Amoxicillin in the Non-Surgical Treatment of Peri-Implantitis: 1 Year Follow-Up. Braz. Oral Res..

[60] Mills M.P., Rosen P.S., Chambrone L., Greenwell H., Kao R.T., Klokkevold P.R., McAllister B.S., Reynolds M.A., Romanos G.E., Wang H.-L. (2018). American Academy of Periodontology Best Evidence Consensus Statement on the Efficacy of Laser Therapy Used Alone or as an Adjunct to Non-Surgical and Surgical Treatment of Periodontitis and Peri-Implant Diseases. J. Periodontol..

[61] Berglundh T., Wennström J.L., Lindhe J. (2018). Long-Term Outcome of Surgical Treatment of Peri-Implantitis. A 2-11-Year Retrospective Study. Clin. Oral Implant. Res..

[62] Cha J.K., Lee J.S., Kim C. (2019). Surgical Therapy of Peri-Implantitis with Local Minocycline: A 6-Month Randomized Controlled Clinical Trial. J. Dent. Res..

[63] Bispo Júnior J.P. (2022). Social Desirability Bias in Qualitative Health Research. Rev. Saude Publica.

